# Supplementary material for: Processing Hundreds of SARS-CoV-2 Samples with an In-House PCR-Based Method without Robotics
Source: Viruses. 2021 Aug 28;13(9):1712. doi: 10.3390/v13091712 (PMC8473336; doi:10.3390/v13091712)
Supplement: Supplementary file 1 [file viruses-13-01712-s001.zip › viruses-1307681-supplementary.pdf]

viruses

MDPI

Supplementary Materials

Table S1. Validated samples for the JKU in-house system.

			COBAS System		JKU System		
**No.**	**Sample-ID**	**Buffer**	**Cq**	**Result**	**Cq (N1)**	**Cq (N2)**	**Result**
1	62505168	COBAS	16.80	positive	11.11	10.84	positive
2	62511498	NaCl	17.40	positive	19.67	20.64	positive
3	62515602	NaCl	18.42	positive	21.46	21.52	positive
4	6250958827	NaCl	19.07	positive	21.54	21.21	positive
5	62505173	COBAS	20.14	positive	16.91	17.29	positive
6	62505066	COBAS	20.16	positive	17.36	17.84	positive
7	62505453	COBAS	20.49	positive	19.7	20.03	positive
8	62505183	COBAS	20.66	positive	16.12	16.62	positive
9	62521008	NaCl	21.00	positive	22.53	22.5	positive
10	6250943827	NaCl	21.03	positive	21.43	21.63	positive
11	62505679	COBAS	21.85	positive	20.97	21.61	positive
12	62506522	COBAS	22.70	positive	16.18	16.96	positive
13	62517237	NaCl	23.00	positive	27.19	26.95	positive
14	62513328	COBAS	23.43	positive	20.88	21.4	positive
15	62517464	COBAS	23.59	positive	21.94	22.73	positive
16	62505456	COBAS	23.66	positive	18.69	23.28	positive
17	62505255	COBAS	23.76	positive	21.61	22.32	positive
18	62505202	COBAS	23.83	positive	20.8	21.15	positive
19	62504987	COBAS	23.85	positive	21.97	22.64	positive
20	62511482	NaCl	24.00	positive	25.9	27.32	positive
21	62504863	COBAS	24.44	positive	21.05	21.73	positive
22	62524349	COBAS	24.63	positive	26.32	27.68	positive
23	62504989	COBAS	24.93	positive	22.86	23.46	positive
24	62520965	NaCl	25.00	positive	25.18	25.13	positive
25	62505068	COBAS	25.13	positive	22.02	22.75	positive
26	62524029	NaCl	25.35	positive	28.51	28.6	positive
27	62511485	NaCl	26.00	positive	30.16	30.23	positive
28	6251206827	COBAS	26.17	positive	24.03	25.68	positive
29	62505059	COBAS	26.19	positive	24.06	24.6	positive
30	6900159327	COBAS	26.89	positive	26.43	27.74	positive
31	62517260	NaCl	27.00	positive	28.12	27.84	positive
32	62516844	NaCl	27.19	positive	28.36	28.28	positive
33	62515653	NaCl	27.48	positive	29.34	30.63	positive
34	62505198	COBAS	27.57	positive	21.87	22.5	positive
35	62513742	NaCl	27.61	positive	26.02	26.32	positive
36	6900159827	COBAS	27.73	positive	25.32	26.39	positive
37	62505219	COBAS	27.84	positive	26.13	27.16	positive
38	62504893	COBAS	27.88	positive	22.81	23.59	positive
39	62518965	COBAS	28.00	positive	31.7	32.66	positive
40	6250927527	NaCl	28.76	positive	36.16	36.81	positive
41	62505672	COBAS	28.85	positive	26.22	27.1	positive
42	62520042	COBAS	28.90	positive	28.64	29.45	positive
43	62505067	COBAS	29.11	positive	26.54	27.34	positive

44	62506529	COBAS	29.26	positive	22.22	22.98	positive
45	6251205627	COBAS	29.58	positive	28.65	29.68	positive
46	62511450	NaCl	29.60	positive	32.25	32.83	positive
47	62519301	NaCl	29.67	positive	30.72	32.17	positive
48	62534743	NaCl	29.77	positive	33.19	34.8	positive
49	62528467	NaCl	29.82	positive	35.54	37.22	positive
50	6250969527	NaCl	29.88	positive	34.6	36.29	positive
51	62520507	NaCl	30.00	positive	35.6	37.06	positive
52	62520549	NaCl	30.00	positive	32.69	34.02	positive
53	62520899	NaCl	30.00	positive	31.28	31.53	positive
54	62519541	NaCl	30.49	positive	30.76	31.53	positive
55	62517116	NaCl	30.70	positive	33.32	33.93	positive
56	62519879	COBAS	30.72	positive	28.51	29	positive
57	62511451	NaCl	31.00	positive	36.63	37.45	positive
58	62520555	NaCl	31.00	positive	.	37.06	inconclusive
59	62528415	NaCl	31.16	positive	.	39.11	inconclusive
60	62505666	COBAS	31.30	positive	29.37	30	positive
61	62524355	COBAS	31.46	positive	32.73	34.23	positive
62	6250980827	NaCl	31.66	positive	.	.	negative
63	62511433	NaCl	32.00	positive			negative
64	62517221	NaCl	32.00	positive	34.83	36.74	positive
65	62517230	NaCl	32.00	positive	.	.	negative
66	62520603	COBAS	32.00	positive	32.11	32.53	positive
67	6251205827	COBAS	32.00	positive	32.08	33.83	positive
68	62505326	COBAS	32.20	positive	30.18	30.95	positive
69	6251207027	COBAS	32.30	positive	33.04	34.09	positive
70	62515610	NaCl	32.35	positive			negative
71	62511888	NaCl	32.60	positive	38.86	38.76	positive
72	62524685	NaCl	32.90	positive	38.21	.	inconclusive
73	62515633	NaCl	33.00	positive			negative
74	62520081	COBAS	33.00	positive	34.88	37.01	positive
75	62520488	COBAS	33.00	positive	32.2	32.33	positive
76	62524053	NaCl	33.20	positive	36.06	38.71	positive
77	62535117	NaCl	33.25	positive	.	.	negative
78	62505237	COBAS	33.30	positive	30.24	31.15	positive
79	62520472	NaCl	34.00	positive	32.4	31.96	positive
80	62520502	NaCl	34.00	positive	.	36.93	inconclusive
81	62520909	NaCl	34.00	positive	.	.	negative
82	62520982	NaCl	34.00	positive	.	.	negative
83	6900155127	COBAS	34.00	positive	35.81	36.64	positive
84	62534773	NaCl	34.05	positive	38.35	.	inconclusive
85	62534801	NaCl	34.25	positive	.	39.25	inconclusive
86	62528772	NaCl	34.68	positive	35.02	37.03	positive
87	62505191	COBAS	34.84	positive	31.15	32.06	positive
88	62520557	NaCl	35.00	positive	.	.	negative
89	62520866	NaCl	35.00	positive	.	.	negative
90	62528437	NaCl	35.33	positive	.	.	negative
91	6900149127	COBAS	35.33	positive	36.08	38.38	positive
92	62534802	NaCl	35.65	positive	.	.	negative

93	62520211	COBAS	35.72	positive	30.68	31.06	positive
94	80272462	COBAS	35.96	positive	37.86	39.2	positive
95	62520130	COBAS	36.00	positive	36.63	39.33	positive
96	62534234	NaCl	36.00	positive	.	.	negative
97	6900153427	COBAS	36.00	positive	37.24	36.8	positive
98	62519520	NaCl	36.11	positive	34.84	36.32	positive
99	62528419	NaCl	36.29	positive	.	.	negative
100	62534745	NaCl	36.57	positive	.	.	negative
101	62534246	NaCl	36.62	positive	.	45.95	inconclusive
102	62534794	NaCl	36.69	positive	.	.	negative
103	62505263	COBAS	36.93	positive	32.38	33.77	positive
104	62517264	NaCl	37.00	positive	.	.	negative
105	62520508	NaCl	37.00	positive	37.04	39.12	positive
106	62520929	COBAS	37.00	positive	.	.	negative
107	62524024	NaCl	37.06	positive	38.03	39.13	positive
108	62506506	COBAS	37.86	positive	29.63	30.49	positive
109	62520969	NaCl	38.00	positive	.	.	negative
110	62524031	NaCl	38.03	positive	38.53	.	inconclusive
111	6900159427	COBAS	38.04	positive	38.61	38.17	positive
112	62519536	NaCl	38.22	positive	.	.	negative
113	62519510	NaCl	38.23	positive	38.27	.	inconclusive
114	62534752	NaCl	38.62	positive	.	.	negative
115	62506509	COBAS	38.66	positive	.	34.38	inconclusive
116	62534696	COBAS	39.77	positive	.	.	negative
117	62505178	COBAS	40.00	positive	34.97	33.27	positive
118	62513424	COBAS		negative	29.01	30.21	positive
119	62513391	COBAS		negative	31.39	32.39	positive
120	62523**5	COBAS		negative	33.57	35.28	positive
121	62511893	NaCl		negative	34.86	37.55	positive
122	62542141	NaCl		negative	38.64		inconclusive
123	62505732	COBAS		negative	.	43.62	inconclusive
124	62541641	NaCl		negative		41.48	inconclusive
125	62513009	COBAS		negative	.	.	negative
126	62513226	COBAS		negative	.	.	negative
127	62513270	COBAS		negative	.	.	negative
128	62513299	COBAS		negative	.	.	negative
129	62513326	COBAS		negative	.	.	negative
130	62513343	COBAS		negative	.	.	negative
131	62513359	COBAS		negative	.	.	negative
132	62513386	COBAS		negative	.	.	negative
133	62513402	COBAS		negative	.	.	negative
134	62513430	COBAS		negative	.	.	negative
135	69001390	COBAS		negative	.	.	negative
136	69001391	COBAS		negative	.	.	negative
137	69001392	COBAS		negative	.	.	negative
138	69001393	COBAS		negative	.	.	negative
139	69001396	COBAS		negative	.	.	negative
140	69001397	COBAS		negative	.	.	negative
141	69001398	COBAS		negative	.	.	negative

142	62517222	NaCl	negative	.	.	negative
143	62518590	NaCl	negative	.	.	negative
144	62505726	COBAS	negative	.	.	negative
145	62505728	COBAS	negative	.	.	negative
146	62505730	COBAS	negative	.	.	negative
147	62522862	COBAS	negative	.	.	negative
148	62522868	NaCl	negative	.	.	negative
149	62522873	NaCl	negative	.	.	negative
150	62522894	COBAS	negative	.	.	negative
151	62522905	COBAS	negative	.	.	negative
152	62522916	COBAS	negative	.	.	negative
153	62522925	COBAS	negative	.	.	negative
154	62522927	COBAS	negative	.	.	negative
155	62522964	COBAS	negative	.	.	negative
156	62522988	COBAS	negative	.	.	negative
157	62522996	COBAS	negative	.	.	negative
158	62522997	COBAS	negative	.	.	negative
159	62523008	COBAS	negative	.	.	negative
160	62523027	COBAS	negative	.	.	negative
161	62523031	COBAS	negative	.	.	negative
162	62523034	COBAS	negative	.	.	negative
163	62523076	COBAS	negative	.	.	negative
164	62523097	COBAS	negative	.	.	negative
165	62523100	COBAS	negative	.	.	negative
166	62523289	COBAS	negative	.	.	negative
167	62523299	COBAS	negative	.	.	negative
168	62523312	COBAS	negative	.	.	negative
169	62523313	COBAS	negative	.	.	negative
170	62523314	COBAS	negative	.	.	negative
171	62523316	COBAS	negative	.	.	negative
172	62523326	COBAS	negative	.	.	negative
173	6900157027	COBAS	negative	.	.	negative
174	62532907	COBAS	negative	.	.	negative
175	62532936	COBAS	negative	.	.	negative
176	62532968	COBAS	negative	.	.	negative
177	62532971	COBAS	negative	.	.	negative
178	62532972	COBAS	negative	.	.	negative
179	62532985	COBAS	negative	.	.	negative
180	62532988	COBAS	negative	.	.	negative
181	62532993	COBAS	negative	.	.	negative
182	62533002	COBAS	negative	.	.	negative
183	62533004	COBAS	negative	.	.	negative
184	62533033	COBAS	negative	.	.	negative
185	62533036	COBAS	negative	.	.	negative
186	62533039	COBAS	negative	.	.	negative
187	6254993327	COBAS	negative	.	.	negative
188	6255101827	COBAS	negative	.	.	negative
189	6255113727	COBAS	negative	.	.	negative
190	6255116427	NaCl	negative	.	.	negative

191	6255116827	NaCl	negative	.	.	negative
192	6255117427	NaCl	negative	.	.	negative
193	6255117627	NaCl	negative	.	.	negative
194	6255117827	NaCl	negative	.	.	negative
195	6255118027	NaCl	negative	.	.	negative
196	6255118127	NaCl	negative	.	.	negative
197	6255118427	NaCl	negative	.	.	negative
198	6255118527	NaCl	negative	.	.	negative
199	6255118727	NaCl	negative	.	.	negative
200	6255119027	NaCl	negative	.	.	negative
201	6255119227	NaCl	negative	.	.	negative
202	6255119427	NaCl	negative	.	.	negative
203	6255119527	NaCl	negative	.	.	negative
204	6255119627	NaCl	negative	.	.	negative
205	6255119827	NaCl	negative	.	.	negative
206	6255120527	NaCl	negative	.	.	negative
207	6900181727	COBAS	negative	.	.	negative
208	6900181827	COBAS	negative	.	.	negative
209	6900230027	COBAS	negative	.	.	negative
210	6900230827	COBAS	negative	.	.	negative
211	6900231127	COBAS	negative	.	.	negative
212	62541615	NaCl	negative			negative
213	62541616	NaCl	negative			negative
214	62541618	NaCl	negative			negative
215	62541621	NaCl	negative			negative
216	62541624	NaCl	negative			negative
217	62541631	NaCl	negative			negative
218	62541643	NaCl	negative			negative
219	62541656	NaCl	negative			negative

Shown are 219 samples collected in COBAS buffer or NaCl that have been initially measured with the Roche COBAS system and finally analysed for our in-house system (JKU system) using the two virus-specific probes N1 and N2. Samples are classified due to their viral load according to the COBAS system: high viral load, Cq ≤ 30, blue; low viral load, Cq > 30, green; negative, no signal detection, black.

Table S2. Differences in SARS-CoV-2 detection between KUK and JKU for samples with low viral load (Cq > 30).

			COBAS System		JKU System - Replicate 1				JKU System - Replicate 2			
**No.**	**Sample-ID**	**Buffer**	**Cq**	**Result**	**Cq (N1)**	**Cq (N2)**	**Cq (RP)**	**Result**	**Cq (N1)**	**Cq (N2)**	**Cq (RP)**	**Result**
1	62505732	COBAS	n.a.	negative	n.a.	43.62	24.92	inconclu-sive	n.a.	n.a.	24.88	negative
2	62506509	COBAS	38.7	positive	n.a.	34.38	21.16	inconclu-sive	34.94	35.81	21.13	positive
3	62513391	COBAS	n.a.	negative	31.39	32.39	25.79	positive	n.a.	38.27	22.84	inconclu-sive
4	62513424	COBAS	n.a.	negative	29.01	30.21	26.01	positive	n.a.	n.a.	24.83	negative
5	62520929	COBAS	37.0	positive	n.a.	n.a.	27.27	negative	n.a.	n.a.	27.05	negative
6	62523**5	COBAS	n.a.	negative	33.57	35.28	24.62	positive	35.55	36.69	26.31	positive
7	62534696	COBAS	39.8	positive	n.a.	n.a.	26.39	negative	n.a.	n.a.	27.11	negative
8	6900210727	COBAS	n.a.	negative	39.62	38.66	25.25	positive				
1	62517264	NaCl	37.0	positive	n.a.	n.a.	32.23	negative	n.a.	n.a.	32.93	negative
2	62517230	NaCl	32.0	positive	n.a.	n.a.	28.94	negative				
3	62520969	NaCl	38.0	positive	n.a.	n.a.	29.4	negative	n.a.	n.a.	28.34	negative
4	62520909	NaCl	34.0	positive	n.a.	n.a.	33.95	negative	n.a.	n.a.	33.68	negative
5	62520866	NaCl	35.0	positive	n.a.	n.a.	33.52	negative	35.83	n.a.	33	inconclu-sive
6	62520565	NaCl	33.0	positive	n.a.	n.a.	n.a.	invalid	n.a.	n.a.	40.3	negative
7	62520555	NaCl	31.0	positive	n.a.	37.06	32.97	inconclu-sive	n.a.	34.45	32.52	inconclu-sive
8	62520502	NaCl	34.0	positive	n.a.	36.93	26.92	inconclu-sive	n.a.	n.a.	26.22	negative
9	62520982	NaCl	34.0	positive	n.a.	n.a.	34.68	negative	37.33	38.43	32.79	positive
10	62520557	NaCl	35.0	positive	n.a.	n.a.	37.57	negative	38.61	n.a.	33.43	inconclu-sive
11	62528419	NaCl	36.3	positive	n.a.	n.a.	28.38	negative	37.26	37.83	27.77	positive
12	62524031	NaCl	38.0	positive	38.53	n.a.	29.17	inconclu-sive				
13	62528437	NaCl	35.3	positive	n.a.	n.a.	27.72	negative	n.a.	n.a.	28.56	negative
14	62528415	NaCl	31.2	positive	n.a.	39.11	27.26	inconclu-sive	33.84	34.65	28	positive
15	62524685	NaCl	32.9	positive	38.21	n.a.	29.45	inconclu-sive	37.03	39.07	28.54	positive
16	62512003	NaCl	37.0	positive	n.a.	n.a.	32.19	negative	n.a.	n.a.	32.43	negative
17	62516229	NaCl	38.0	positive	n.a.	n.a.	31.73	negative	n.a.	n.a.	31.06	negative
18	62534246	NaCl	36.6	positive	n.a.	45.95	27.39	inconclu-sive	n.a.	n.a.	27.72	negative
19	62534234	NaCl	36.0	positive	n.a.	n.a.	28.59	negative	n.a.	n.a.	28.77	negative
20	62534745	NaCl	36.6	positive	n.a.	n.a.	28.34	negative	n.a.	n.a.	28.62	negative
21	62519536	NaCl	38.2	positive	n.a.	n.a.	34.14	negative	n.a.	n.a.	34.65	negative
22	62519510	NaCl	38.2	positive	38.27	n.a.	28.27	inconclu-sive	n.a.	n.a.	29.01	negative
23	62534802	NaCl	35.7	positive	n.a.	n.a.	31.66	negative	n.a.	n.a.	30.35	negative
24	62535117	NaCl	33.3	positive	n.a.	n.a.	33.48	negative	n.a.	n.a.	34.61	negative
25	62534801	NaCl	34.3	positive	n.a.	39.25	31.08	inconclu-sive	n.a.	n.a.	30.76	negative
26	62534794	NaCl	36.7	positive	n.a.	n.a.	32.57	negative	n.a.	n.a.	32.02	negative
27	62534752	NaCl	38.6	positive	n.a.	n.a.	31.57	negative	n.a.	n.a.	31.11	negative
28	62534773	NaCl	34.1	positive	38.35	n.a.	32.11	inconclu-sive	n.a.	39.95	33.25	inconclu-sive

Table S3. Ring trial reflecting the sensitivity and specificity of our assay.

	COBAS System		JKU System Replicate 1 (Luna)			JKU System Replicate 2 (TaqMan)		
**Type**	**Cq**	**Result**	**Cq (N1)**	**Cq (N2)**	**Result**	**Cq (N1)**	**Cq (N2)**	**Result**
ring trial 1	22.18	positive	26.38	27.05	positive	27.23	28.52	positive
ring trial 2	n.a.	negative	n.a.	n.a.	negative	n.a.	n.a.	negative
ring trial 3	31.38	positive	35.48	35.89	positive	36.9	37.77	positive
ring trial 4	25.45	positive	29.24	30.27	positive	30.26	31.77	positive
ring trial 5	n.a.	negative	n.a.	n.a.	negative	n.a.	n.a.	negative
ring trial 6	29.01	positive	32.88	34.25	positive	33.56	35.42	positive
ring trial 7	n.a.	negative	n.a.	n.a.	negative	n.a.	n.a.	negative
RNA control			20.1	20.5		20.75	19.93	
NTC			n.a.	n.a.		n.a.	n.a.	

Seven samples were included in the ring trial and have been measured by the COBAS system and our system (JKU system) in two replicates using the two RT-qPCR kits, Luna and TaqMan, for one of the replicated measurements each. As RNA control, 10^5 copies of the Twist Synthetic SARS-CoV-2 RNA was used, and one reaction was performed for each target without adding a PCR template (no-template control, NTC).

Table S4. Technical sensitivity and reproducibility using SARS-CoV-2 RNA control.

		RNA control					
**RNA Copies**	**Replicates**	**Cq (N1)**	**Average Cq**	**Stdev**	**Cq (N2)**	**Average Cq**	**Stdev**
10^2	4×	35.35	35.44	0.47	36.05	35.77	0.27
10^2		34.98			35.87		
10^2		35.32			35.4		
10^2		36.1			35.76		
10^3	4×	31.9	32.04	0.20	32.41	32.44	0.17
10^3		31.89			32.57		
10^3		32.31			32.21		
10^3		32.06			32.55		
10^4	4×	28.41	28.49	0.15	29.03	28.76	0.19
10^4		28.72			28.71		
10^4		28.43			28.71		
10^4		28.41			28.58		
10^5	8×	24.95	24.98	0.16	24.83	24.88	0.26
10^5		25.06			24.92		
10^5		25.22			24.57		
10^5		24.81			24.6		
10^5		24.74			24.87		
10^5		24.95			25.12		
10^5		25.14			24.8		
10^5		24.97			25.36		
**R2**		0.9966			0.9968		
**Efficiency (E-slope)**		88%			93%		

Different dilutions of RNA positive control have been used for RT-qPCR in four or eight replicated measurements.

Table S5. Sensitivity limits of two patient samples.

Dilution	Buffer	Cq (N1)	Average Cq	Stdev	Cq (N2)	Average Cq	Stdev	Cq (RP)	Average Cq	Stdev	Result
**Sample 1 (#62505679)**											
1:1	COBAS	21.20	21.26	0.08	22.18	22.14	0.06	27.00	27.04	0.05	positive
1:1	COBAS	21.31			22.10			27.07			positive
1:10	COBAS	25.20	25.23	0.04	25.96	25.95	0.02	31.01	31.10	0.12	positive
1:10	COBAS	25.26			25.93			31.18			positive
1:100	COBAS	30.47	30.30	0.25	31.13	31.20	0.10	37.10	37.25	0.21	positive
1:100	COBAS	30.12			31.27			37.39			positive
1:1000	COBAS	34.51	34.39	0.18	35.78	33.82	2.78	40.07	39.51	0.79	positive
1:1000	COBAS	34.26			31.85			38.95			positive
**R2**		0.97			0.94			0.99			
**Efficiency (E-slope)**		70%			77%			68%			
**Sample 2 (#62505672)**											
**Dilution**	**Buffer**	**Cq (N1)**	**Average Cq**	**Stdev**	**Cq (N2)**	**Average Cq**	**Stdev**	**Cq (RP)**	**Average Cq**	**Stdev**	**Result**
1:1	COBAS	26.35	26.30	0.08	27.10	27.13	0.04	23.87	23.56	0.44	positive
1:1	COBAS	26.24			27.15			23.25			positive
1:10	COBAS	29.66	29.75	0.12	31.51	31.08	0.62	26.15	26.07	0.11	positive
1:10	COBAS	29.83			30.64			25.99			positive
1:100	COBAS	33.79	33.56	0.33	34.76	34.73	0.04	30.36	30.17	0.27	positive
1:100	COBAS	33.32			34.70			29.98			positive
1:1000	COBAS	37.46	37.46		39.34	39.22	0.17	33.73	34.03	0.42	positive
1:1000	COBAS	n.a.			39.10			34.33			inconclu-sive
**R2**		0.9867			0.99			0.99			
**Efficiency (E-slope)**		91%			78%			86%			

Shown is the technical sensitivity and reproducibility using two patient samples in COBAS lysis buffer that have been diluted in a series of 1:10 after extracting viral RNA. For each diluted RNA sample two RT-qPCR replicates were performed.

Table S6. Replicated measurements.

			COBAS System		JKU System - Replicate 1			JKU System - Replicate 2		
No.	Sample-ID	Buffer	Cq	Result	Cq (N1)	Cq (N2)	Result	Cq (N1)	Cq (N2)	Result
1	62505453	COBAS	20.49	positive	19.7	20.03	positive	20.19	20.61	positive
2	6250976427	COBAS	20.93	positive	23.01	24.13	positive	20.21	20.55	positive
3	62521008	NaCl	21	positive	22.53	22.5	positive	22.09	22.51	positive
4	62520127	PBS	23.45	positive	24.94	25.16	positive	24.72	24.9	positive
5	62517464	COBAS	23.59	positive	21.94	22.73	positive	24.27	25.19	positive
6	62520965	NaCl	25	positive	25.18	25.13	positive	25.45	25.51	positive
7	62517260	NaCl	27.00	positive	28.12	27.84	positive	29.83	29.77	positive
8	62516844	NaCl	27.19	positive	28.36	28.28	positive	30.11	30.22	positive
9	62518965	COBAS	28	positive	31.7	32.66	positive	32.72	34.02	positive
10	62520042	COBAS	28.9	positive	28.64	29.45	positive	28.4	28.67	positive
11	62523536	PBS	29.3	positive	31.56	31.87	positive	31	31.56	positive
12	62519301	NaCl	29.67	positive	30.72	32.17	positive	31.31	32.47	positive
13	62534743	NaCl	29.77	positive	33.19	34.8	positive	33.99	35.44	positive
14	62528467	NaCl	29.82	positive	35.54	37.22	positive	32.4	34.3	positive
15	62529629	PBS	29.99	positive	32.05	33.15	positive	32.45	33.4	positive
16	62520899	NaCl	30	positive	31.28	31.53	positive	29.75	30.17	positive
17	62520549	NaCl	30	positive	32.69	34.02	positive	33.18	33.06	positive
18	62520507	NaCl	30	positive	35.6	37.06	positive	42.17	36.08	positive
19	62519541	NaCl	30.49	positive	30.76	31.53	positive	31.5	32.94	positive
20	62517116	NaCl	30.70	positive	33.32	33.93	positive	35.73	36.92	positive
21	62519879	COBAS	30.72	positive	28.51	29	positive	29.49	29.6	positive
22	62520555	NaCl	31	positive	n.a.	37.06	inconclusive	n.a.	34.45	inconclusive
23	62528415	NaCl	31.16	positive	n.a.	39.11	inconclusive	33.84	34.65	positive
24	62524691	NaCl	31.43	positive	34.71	35.57	positive	33.39	33.69	positive
25	62520603	COBAS	32	positive	32.11	32.53	positive	31.69	32.09	positive
26	62505326	COBAS	32.20	positive	30.18	30.95	positive	30.39	31.98	positive
27	62524685	NaCl	32.9	positive	38.21	n.a.	inconclusive	37.03	39.07	positive
28	62520081	COBAS	33	positive	34.88	37.01	positive	37.3	37.91	positive
29	62520488	COBAS	33	positive	32.2	32.33	positive	32.57	32.61	positive
30	62520565	NaCl	33	positive	n.a.	n.a.	invalid	n.a.	n.a.	negative
31	62535117	NaCl	33.25	positive	n.a.	n.a.	negative	n.a.	n.a.	negative
32	62524692	NaCl	33.49	positive	34.94	34.98	positive	34.94	34.98	positive
33	6900155127	COBAS	34	positive	35.81	36.64	positive	33.75	37.63	positive
34	62520909	NaCl	34	positive	n.a.	n.a.	negative	n.a.	n.a.	negative
35	62520502	NaCl	34	positive	n.a.	36.93	inconclusive	n.a.	n.a.	negative
36	62520982	NaCl	34	positive	n.a.	n.a.	negative	37.33	38.43	positive
37	62520472	NaCl	34	positive	32.4	31.96	positive	33.21	32.76	positive
38	62534773	NaCl	34.05	positive	38.35	n.a.	inconclusive	n.a.	39.95	inconclusive

39	62534801	NaCl	34.25	positive	n.a.	39.25	inconclu-sive	n.a.	n.a.	negative
40	62520866	NaCl	35	positive	n.a.	n.a.	negative	35.83	n.a.	inconclu-sive
41	62520557	NaCl	35	positive	n.a.	n.a.	negative	38.61	n.a.	inconclu-sive
42	690014912 7	COBAS	35.33	positive	36.08	38.38	positive	37.86	40.22	positive
43	62528437	NaCl	35.33	positive	n.a.	n.a.	negative	n.a.	n.a.	negative
44	62534802	NaCl	35.65	positive	n.a.	n.a.	negative	n.a.	n.a.	negative
45	62520211	COBAS	35.72	positive	30.68	31.06	positive	30.83	31.31	positive
46	80272462	COBAS	35.96	positive	37.86	39.2	positive	38.18	n.a.	inconclu-sive
47	62520130	COBAS	36	positive	36.63	39.33	positive	n.a.	n.a.	negative
48	690015342 7	COBAS	36	positive	37.24	36.8	positive	40.22	n.a.	inconclu-sive
49	62534234	NaCl	36	positive	n.a.	n.a.	negative	n.a.	n.a.	negative
50	62519520	NaCl	36.11	positive	34.84	36.32	positive	38.56	40.47	positive
51	62528419	NaCl	36.29	positive	n.a.	n.a.	negative	37.26	37.83	positive
52	62534745	NaCl	36.57	positive	n.a.	n.a.	negative	n.a.	n.a.	negative
53	62534246	NaCl	36.62	positive	n.a.	45.95	inconclu-sive	n.a.	n.a.	negative
54	62534794	NaCl	36.69	positive	n.a.	n.a.	negative	n.a.	n.a.	negative
55	62520929	COBAS	37	positive	n.a.	n.a.	negative	n.a.	n.a.	negative
56	62517264	NaCl	37.00	positive	n.a.	n.a.	negative	n.a.	n.a.	negative
57	62520508	NaCl	37	positive	37.04	39.12	positive	n.a.	39.05	inconclu-sive
58	62512003	NaCl	37	positive	n.a.	n.a.	negative	n.a.	n.a.	negative
59	62524024	NaCl	37.06	positive	38.03	39.13	positive	36.22	37.09	positive
60	62527697	NaCl	37.9	positive	37.49	40.18	positive	37.1	40.17	positive
61	62516229	NaCl	37.96	positive	n.a.	n.a.	negative	n.a.	n.a.	negative
62	62520969	NaCl	38	positive	n.a.	n.a.	negative	n.a.	n.a.	negative
63	62519536	NaCl	38.22	positive	n.a.	n.a.	negative	n.a.	n.a.	negative
64	62519510	NaCl	38.23	positive	38.27	n.a.	inconclu-sive	n.a.	n.a.	negative
65	62534752	NaCl	38.62	positive	n.a.	n.a.	negative	n.a.	n.a.	negative
66	62506509	COBAS	38.66	positive	n.a.	34.38	inconclu-sive	34.94	35.81	positive
67	62534696	COBAS	39.77	positive	n.a.	n.a.	negative	n.a.	n.a.	negative
68	62505732	COBAS	n.a.	negative	n.a.	43.62	inconclu-sive	n.a.	n.a.	negative
69	62513391	COBAS	n.a.	negative	31.39	32.39	positive	n.a.	38.27	inconclu-sive
70	62513424	COBAS	n.a.	negative	29.01	30.21	positive	n.a.	n.a.	negative
71	690015702 7	COBAS	n.a.	negative	n.a.	n.a.	negative	n.a.	n.a.	negative
72	62522905	COBAS	n.a.	negative	n.a.	n.a.	negative	n.a.	n.a.	negative
73	62522916	COBAS	n.a.	negative	n.a.	n.a.	negative	n.a.	n.a.	negative
74	62523008	COBAS	n.a.	negative	n.a.	n.a.	negative	n.a.	n.a.	negative
75	62523031	COBAS	n.a.	negative	n.a.	n.a.	negative	39.16	39.7	positive

76	62523313	COBAS	n.a.	negative	n.a.	n.a.	negative	n.a.	n.a.	negative
77	62523299	COBAS	n.a.	negative	n.a.	n.a.	negative	n.a.	n.a.	negative
78	62523076	COBAS	n.a.	negative	n.a.	n.a.	negative	n.a.	n.a.	negative
79	62523**5	COBAS	n.a.	negative	33.57	35.28	positive	35.55	36.69	positive
80	62523314	COBAS	n.a.	negative	n.a.	n.a.	negative	n.a.	n.a.	negative
81	62522894	COBAS	n.a.	negative	n.a.	n.a.	negative	n.a.	n.a.	negative
82	62532907	COBAS	n.a.	negative	n.a.	n.a.	negative	n.a.	n.a.	negative
83	62533033	COBAS	n.a.	negative	n.a.	n.a.	negative	n.a.	n.a.	negative
84	62532972	COBAS	n.a.	negative	n.a.	n.a.	negative	n.a.	n.a.	negative
85	62532968	COBAS	n.a.	negative	n.a.	n.a.	negative	n.a.	n.a.	negative
86	6900181827	COBAS	n.a.	negative	n.a.	n.a.	negative	n.a.	n.a.	negative
87	62533002	COBAS	n.a.	negative	n.a.	n.a.	negative	n.a.	n.a.	negative
88	62532988	COBAS	n.a.	negative	n.a.	n.a.	negative	n.a.	n.a.	negative
89	62532971	COBAS	n.a.	negative	n.a.	n.a.	negative	n.a.	n.a.	negative
90	62532985	COBAS	n.a.	negative	n.a.	n.a.	negative	n.a.	n.a.	negative
91	62532993	COBAS	n.a.	negative	n.a.	n.a.	negative	n.a.	n.a.	negative
92	62533039	COBAS	n.a.	negative	n.a.	n.a.	negative	n.a.	n.a.	negative
93	62533004	COBAS	n.a.	negative	n.a.	n.a.	negative	n.a.	n.a.	negative
94	62533036	COBAS	n.a.	negative	n.a.	n.a.	negative	n.a.	n.a.	negative
95	62532936	COBAS	n.a.	negative	n.a.	n.a.	negative	n.a.	n.a.	negative
96	6900181727	COBAS	n.a.	negative	n.a.	n.a.	negative	n.a.	n.a.	negative
97	62522873	NaCl	n.a.	negative	n.a.	n.a.	negative	n.a.	n.a.	negative
98	62522868	NaCl	n.a.	negative	n.a.	n.a.	negative	n.a.	n.a.	negative

98 patient samples collected in COBAS buffer, NaCl or PBS were initially measured by the COBAS system and analysed with our system (JKU system) in two replicated measurements. The samples were grouped in high viral load ($Cq \leq 30$, blue), low viral load ($Cq > 30$, green) and not detected (no Cq, black) based on the COBAS measurements.

Table S7. Limit of detection and RNA extraction efficiency.

RNA Extraction Efficiency						
**Sample type**	**Sample-ID**	**Dilution**	**Buffer**	**Cq (N1)**	**Cq (N2)**	**Cq (RP)**
Pool of negative COBAS samples + 1:10 dilution of RNA control	#62523312 #62523289 #62522964 #62522996 #62523100	10^6 RNA copies/reaction	COBAS	27.13	27.05	23.21
		10^5 RNA copies/reaction	COBAS	30.74	30.67	23.68
		10^4 RNA copies/reaction	COBAS	33.6	33.94	23.96
		10^3 RNA copies/reaction	COBAS	37	37.75	23.95
		10^2 RNA copies/reaction	COBAS	n.a.	40.49	23.86
1:5 dilution series of positive COBAS sample	Sample 1 #62524349	1:1	COBAS	24.89	25.91	27.22
		1:5	buffer 6	27.12	28.47	29.3
		1:25	buffer 6	30.05	31.27	31.85
		1:125	buffer 6	32.82	33.96	35.38
		1:625	buffer 6	34.86	36.74	37.8
1:5 dilution series of positive COBAS sample	Sample 2 #6900159327	1:1	COBAS	26.37	27.13	27.28
		1:5	buffer 6	28.21	29.11	27.77
		1:25	buffer 6	30.72	31.68	30.48
		1:125	buffer 6	34.1	35.01	33.47
		1:625	buffer 6	38.02	38.32	37.03

Different copies of positive RNA control were added to a pool of negatively tested patient samples before RNA extraction. In addition, two positive patient samples collected in COBAS buffer were diluted in a 1:5 dilution series using buffer 6 before RNA extraction. For all samples, the complete protocol was performed (RNA extraction + RT-qPCR) to detect the N1, N2 and RP targets.

Table S8. Sample stability.

		Day 1		Day 1			Day 3	
**Sample**	**Buffer**	**Cq (CO-BAS)**	**Cq (N2)**	**Cq (E-Sar-beco)**	**Result**	**Cq (N2)**	**Cq (E-Sar-beco)**	**Result**
1	PBS	n.a.	n.a.	n.a.	negative	n.a.	n.a.	negative
2	PBS	n.a.	n.a.	n.a.	negative	n.a.	n.a.	negative
3	PBS	n.a.	n.a.	n.a.	negative	n.a.	n.a.	negative
4	PBS	n.a.	n.a.	n.a.	negative	n.a.	n.a.	negative
5	PBS	n.a.	n.a.	n.a.	negative	n.a.	n.a.	negative
6	PBS	n.a.	n.a.	n.a.	negative	n.a.	n.a.	negative
7	PBS	n.a.	n.a.	n.a.	negative	n.a.	n.a.	negative
8	PBS	n.a.	n.a.	n.a.	negative	n.a.	n.a.	negative
9	PBS	n.a.	n.a.	n.a.	negative	n.a.	n.a.	negative
10	PBS	n.a.	n.a.	n.a.	negative	n.a.	n.a.	negative
11	PBS	n.a.	n.a.	n.a.	negative	n.a.	n.a.	positive
12	PBS	n.a.	n.a.	n.a.	negative	n.a.	n.a.	negative
13	PBS	n.a.	n.a.	n.a.	negative	n.a.	n.a.	negative
14	PBS	n.a.	n.a.	n.a.	negative	n.a.	n.a.	negative
15	PBS	n.a.	n.a.	n.a.	negative	n.a.	n.a.	negative
16	PBS	17	16.89	17.5	positive	19.01	19	positive
17	PBS	18.63	19.91	21.57	positive	20.6	21.08	positive
18	PBS	19.99	23.84	23.81	positive	24.38	24.73	positive
19	PBS	20.41	21.5	22.73	positive	22.96	24.13	positive
20	PBS	21.77	24.82	26.01	positive	26.22	27.01	positive
21	PBS	24.43	26.28	26.45	positive	28.41	28.47	positive
22	PBS	26.01	27.74	28.94	positive	28.19	28.37	positive
23	PBS	26.97	31.73	31.71	positive	33.14	32.75	positive
24	PBS	27.36	29.29	29.4	positive	30.37	30.48	positive
25	PBS	27.88	34.21	34.67	positive	34.33	35.32	positive
26	PBS	28.9	32.1	32.44	positive	33.29	33.4	positive
27	PBS	29.18	21.47	22.36	positive	22.38	23.1	positive
28	PBS	30.48	31.11	31.52	positive	32.39	33.46	positive
29	PBS	30.61	33.42	34.14	positive	34.33	35.27	positive
30	PBS	30.8	33.47	33.32	positive	34.79	35.07	positive
31	PBS	32.92	n.a.	n.a.	negative	n.a.	38.12	inconclusive
32	PBS	33.7	39.03	n.a.	inconclusive	n.a.	n.a.	negative
33	PBS	33.87	n.a.	n.a.	negative	n.a.	n.a.	negative
34	PBS	35.19	n.a.	38.47	inconclusive	n.a.	n.a.	negative
35	PBS	38.21	n.a.	n.a.	negative	n.a.	n.a.	negative

35 patient sample collected in PBS buffer have been pre-analysed by the COBAS system and measured with our in-house system at day 1 and 3 using the virus-specific probes N2 and E-Sarbeco. Based on the COBAS results we classified the samples in high viral load ($Cq \leq 30$, blue), low viral load ($Cq > 30$, green) and negative (not detected, black).

Table S9. RP_2 validation data.

RP_2 Validation Data								
ID	sample	Cq COBAS	N2 JKU	E-Sarbeco JKU	RP w RT JKU	RP w/o RT JKU	RP_2 w RT JKU	RP_2 w/o RT JKU
1	9411437727	n.a.	n.a.	n.a.	28	27.74	35.4	n.a.
2	9411437527	n.a.	n.a.	n.a.	26.39	26.47	36.05	n.a.
3	9411437027	n.a.	n.a.	n.a.	29.52	29.61	37.02	n.a.
4	9411439127	n.a.	n.a.	n.a.	27.52	26.32	35.41	n.a.
5	9411439027	n.a.	n.a.	n.a.	29.43	28.75	n.a.	n.a.
6	8028024227	n.a.	n.a.	n.a.	33.51	32.69	n.a.	n.a.
7	9411435127	n.a.	n.a.	n.a.	27.82	27.75	n.a.	n.a.
8	9411435327	n.a.	n.a.	n.a.	28.5	28.49	36.28	n.a.
9	9411435027	n.a.	n.a.	n.a.	31.42	31.63	n.a.	n.a.
10	9411437327	n.a.	n.a.	n.a.	26.68	26.78	n.a.	n.a.
11	9411437227	n.a.	n.a.	n.a.	30.63	30.81	38.14	n.a.
12	9411438827	n.a.	n.a.	n.a.	32.24	32.33	n.a.	n.a.
13	6900500827	n.a.	n.a.	n.a.	32.66	32.1	36.86	n.a.
14	6267461427	n.a.	n.a.	n.a.	28.01	28.28	33.59	n.a.
15	6267474927	n.a.	n.a.	n.a.	29.9	29.57	36.96	n.a.
16	9411408927	30.48	31.11	31.52	27.66	28.01	34.33	n.a.
17	7331685527	38.21	n.a.	n.a.	28.22	27.65	n.a.	n.a.
18	7331711527	28.9	32.1	32.44	31.91	32.85	n.a.	n.a.
19	6267121327	20.41	21.5	22.73	27.94	27.99	n.a.	n.a.
20	9411408827	17	16.89	17.5	27.11	27.1	n.a.	n.a.

Detailed Standard operation Procedure (SOP)

1. Test Material

Naso-pharyngeal swabs from patients in different buffers (COBAS, NaCl, and PBS).

2. Specimen analysis

2.1. Equipment

Regular calibration/maintenance should be performed annually for pipettes and BSC hoods.

- 2× PCR workstation with filtered airflow and UV-light Search (Thermo Scientific MSC-Advantage™ Class II Biological Safety Cabinets; 1.8 m)
- Magnetic Stand 96 well plate (Thermo Fisher, #AM10027) - for plate extractions
- Magnetic Particle Concentrator (MPC) DynaMagnet (Thermo Scientific, # 12321D)
- Two Bio-Rad CFX384™Real-Time System type C1000 thermocycler (Bio-Rad)
- Combispin FVL 2400 with Vortex (Hartenstein)
- Plate centrifuge: Megafuge 16R (Thermo Scientific)
- Rainin Single channel LTS 0.2–10 µL, 2–20 µL, 20–200 µL and 100–1000 µL Pipettes (Mettler Toledo)
- Rainin electronic multichannel E-12 200XLS (Mettler Toledo)
- Rainin E4 LTS 8 channel adjustable 1200 µL pipette (Mettler Toledo)
- Rainin electronic multichannel E-12 1200XLS (Mettler Toledo)
- Rainin electronic multichannel E-12 20XLS (Mettler Toledo)
- Snijders Scientific Ultra Low Freezer –86 °C (evosafe series VF720-86) (Bartelt)
- (PX1 PCR plate sealer (Bio-Rad))

2.2. Software

Bio-Rad CFX Manager 3.1

2.3. Reagents and Chemicals

2.3.1. RNA extraction

SeraSil-Mag (recently renamed to Sera-Xtracta Virus/Pathogen Kit); (Cytiva, #28990028; LOT: LS-039878)

Other reagents that are not provided in kit:

Ethanol absolute (absolute 99%, for molecular biology; #A80752500PE; Bartelt)

Nuclease-free water (for molecular biology; RNase and DNase free, not DEPC-treated; #AM9932; Thermo Fisher)

2.3.2. RT-qPCR

**authorized to be used for COVID-19 testing by the Centers for Disease Control and Prevention approved by the FDA for emergency use; see https://www.cdc.gov/coronavirus/2019-ncov/php/testing.html, accessed on 9 June 2021)

**Primers and probes: 2019-nCoV CDC EUA Kit (IDT, #10006606; LOT: 0000510344)

**TaqMan™ Fast Virus 1-Step Master Mix (Thermo Fisher, #4444432; LOT: 889999)

Luna® Probe One-Step RT-qPCR Kit (NEB, #E3007; LOT: 10072952,)

Positive control: Twist Synthetic SARS-CoV-2 RNA Control 1, Control (102019) (Twist Bioscience, #MT007544.1; LOT: 2000002381)

2.4. Primer/Probes

Table SOP1: Primer/probe sets N1, N2 and RP suggested by CDC.

**COVID-19 Specific Primers/Probes** (2019-nCoV CDC EUA Kit; IDT)		
2019-nCoV_N1-F	N-gene	GACCCCAAAATCAGCGAAAT
2019-nCoV_N1-R	N-gene	TCTGGTTACTGCCAGTTGAATCTG
2019-nCoV_N1-P	N-gene	FAM-ACCCCGCATTACGTTTGGTG-GACC-BHQ1
2019-nCoV_N2-F	N-gene	TTACAAACATTGGCCGCAAA
2019-nCoV_N2-R	N-gene	GCGCGACATTCCGAAGAA
2019-nCoV_N2-P	N-gene	FAM-ACAATTT-GCCCCCAGCGCTTCAG-BHQ1
**Internal Control Primers (RPP30, RNase P)**		
RP-F	RNase P	AGATTTGGACCTGCGAGCG
RP-R	RNase P	GAGCGGCTGTCTCCACAAGT
RP-P	RNase P	FAM–TTCTGAC-CTGAAGGCTCTGCGCG–BHQ1

2.5. Further Consumables

Optical 384-well plates (Bio-Rad, #HSP3865)
Adhesive optical clear RT-PCR plate seals (Bio-Rad, #MSB1001)
Film Sealing Roller for PCR Plates (Bio-Rad)
Clear adhesive film (Thermo Fisher, #4306311 or GE Healthcare, #7704-0001)
Filter tips:

- Rainin LTS, RTL 10F 0.5–20 µL (Mettler Toledo)
- Rainin LTS, RTL 200F 20–200 µL (Mettler Toledo)
- Rainin RT LTS 1200µL F 768A/8 (Mettler Toledo)
- Rainin LTS, RTL 100–1000F µL (Mettler Toledo)

1.6 mL low-binding tubes DNase/RNase free (Biozym, #710176 or Lab Consulting, #28.5101500C-1)
Deep bottom well plates 96W 2ML UNP NPP 25/PK (GE Healthcare, #7701-5200 or Thermo Fisher, #95040450)
Elution plate U-bottom (Macherey-Nagel by GE Healthcare, #740486.24)
Nuclease free Pipetting reservoirs (Channel Mate; StarLab; E1306-2510)
Serological pipettes (Greiner Bio-One, #607180)
(Easy Pierce Heat Sealing Foil 20µm (ThermoFisher, #AB-1720))

3. Implementation

3.1. Patient Sample Preparation

The naso-pharyngeal swabs are taken in lysis buffer or virus stabilizing buffer. The data is pseudo-anonymized by a barcode used to label each sample. The barcode is linked to the patient's data in the mainframe of the hospital.

3.2. General Preparations

For general preparations we followed the guidelines by the Centers for Disease Control and Prevention approved by the FDA for emergency use; see https://www.cdc.gov/coronavirus/2019-ncov/php/testing.html, accessed on 9 June 2021).

3.2.1. Primer and Probe Preparation:

Upon receipt, store dried primers and probes at 2–8 °C.

1. Precautions: These reagents should only be handled in a clean area and stored at appropriate temperatures (see below) in the dark. Freeze-thaw cycles should be avoided. Maintain cold when thawed (kept on a cold block at all times during preparation and use)

2. If probes are shipped as a lyophilized powder, using an aseptic technique, suspend dried reagents in nuclease-free water and allow to rehydrate for 15 min at room temperature in the dark, then prepare the primer/probe mix. Currently probes are shipped in pre-mixed solution.
3. Mix gently and aliquot primers/probe in 300 μL volumes into 5 pre-labeled tubes. Store a single aliquot of primers/probe at 2–8 °C in the dark. Do not refreeze (stable for up to 4 months). Store the remaining aliquots at ≤ –20 °C in a non-frost-free freezer, if not used within the next 3–4 weeks.

3.2.2. Synthetic SARS-CoV-2 RNA Control Preparation:

1. Precautions: This reagent should be handled with caution in a dedicated nucleic acid handling area to prevent possible contamination. Freeze-thaw cycles should be avoided. Maintain on ice when thawed.
2. Make single use aliquots (approximately 5 μL) and store at ≤ –70°C. Prepare positive control RNA: Twist Synthetic SARS-CoV-2 RNA control 1 (MT007544.1). The control RNA has a concentration of 10^6 copies/μL. Dilution: 0.8μL + 19.2 μL nuclease-free water = 4 × 10^4 copies/μL; 2.5 μL of the dilution will lead to 10^5 copies/reaction. Prepare a dilution series of 1:10 to finally use $10^{5,}$ 10^4, 10^3 and 10^2 copies/reaction. These dilutions are used as controls for positive N1 and N2 detection. Note that this control is added also to the RP target, which is expected to render a negative result.
3. Thaw a single aliquot (5 μL at 10^6 copies/μL) of a positive control for 4–5 experiments and prepare the dilutions that are kept on ice until adding to the plate. Keep the remaining stock and dilution sample on ice until further usage. The remaining aliquot is stored in the fridge for approximately a week. Do not freeze and thaw again.

3.2.3. No Template Control (NTC)

Sterile, nuclease-free water
Aliquot into volumes of 10 mL
Used to check for contamination during specimen extraction and/or plate set-up

3.2.4. Specimens

Specimens can be stored at 2–8 °C for up to a week after collection if in guanidine-containing buffer.

If a delay in extraction is expected, store specimens at –70 °C or lower.

Extracted nucleic acid is stored at –70 °C or lower.

3.2.5. Safety

Before starting the specimen handling and RNA extraction:

Clean and decontaminate all work surfaces, centrifuges and other equipment (10% bleach, 70% ethanol, or DNA-off (Takara))

Expose Biological Safety Cabinets with UV for 10 min

3.3. Specimen Organization and Preparation

Bar code on the tube with specimen is read and tube is placed on a rack. We recommend depositing swabs using a buffer containing guanidine (COBAS swabs) or our in-house COBAS-like 1× buffer 6. Swabs can also be taken in NaCl or other viral stabilizing collection tubes (e.g. PBS), but these need to be treated with viral inactivation agents achieved for example with the addition of an equivalent volume of 2× buffer 6 (9M Guanidine-Hydrochloride NaCl, 0.1 M Tris-HCl, pH7.4) or buffer 4 (for buffer conditions see Table 6).

3.4. Viral RNA Extraction

For this protocol, the following pipettes can be used:
Rainin electronic multichannel E-12 20XLS
Rainin electronic multichannel E-12 200XLS
Rainin electronic multichannel E-12 1200XLS
Rainin E4 LTS 8-multichannel adjustable 1200 µL

Table SOP2. Overview of programs saved for the Multichannel pipettes.

RNALYSIS	***SN1000***	***TRANSFER***
volume: 570 µL<br>aspirate speed: 5<br>dispense speed: 5	volume: 1000 µL<br>aspirate speed: 5<br>dispense speed: 10	volume: 48 µL<br>aspirate speed: 5<br>dispense speed: 10
WASH	***ELUTE***	***SAMPLE400***
volume: 950 µL<br>aspirate speed: 5<br>dispense speed: 5<br>mix speed: 10<br>mix volume: 750 µL<br>mix cycles: 20	volume: 50 µL<br>aspirate speed: 10<br>dispense speed: 5<br>mix speed: 5<br>mix volume: 30 µL<br>mix cycles: 20	volume: 400 µL<br>aspirate speed: 5<br>dispense speed: 5<br>mix speed: 10<br>mix volume: 700 µL<br>mix cycles: 20

- Prepare reagents
- Prepare **80% ethanol** wash solution.
- **Note**: Prepare fresh 80% ethanol enough for 950 µL per extraction reaction.
- Prepare a **bead stock mixture** of SeraSil-Mag 400 and SeraSil-Mag 700 beads in a 1:1 ratio. Vortex SeraSil-Mag beads thoroughly before each is added to the pre-mixture and then vortex again prior usage. Add this mixture to a pipetting reservoir if multichannels are used for the next steps.
- **Note**: prepare sufficient bead volume for 20 µL bead mixture per reaction.
- Prepare Proteinase K solution by adding 3 mL nuclease-free water to 60 mg Proteinase K material and dissolve properly to have a final solution of 20 mg/mL. Store the Proteinase K solution then at 2–8 °C (stable for 4 months).

Step 1: Lysis and Nucleic Acid Binding

- Add 20 µL of SeraSil-Mag bead mixture to the deep bottom well plates 96-well or 1.6 mL low-binding tubes. This can be done with the Rainin electronic multichannel E-12 20XLS and pipetting reservoir for pipetting out the 96-well plate. Note that each well in these plates holds a volume of 2 mL. There is no need to change tips when pipetting out this master mix.
- **Note**: Prior to adding, vortex the bead mixture carefully
- Add 10 µL of Proteinase K Solution to a well
- Add 570 µL of Binding/Lysis Reagent. This can be done with the Rainin electronic multichannel E-12 1200XLS (select pipette program 'RNALYSIS') and pipetting reservoir.
- **Note**: this buffer is highly viscous and can be foamy. When adding the buffer to the sample, mix carefully by slowly pipetting up/down to avoid multiple bubbles.
- The tubes of the specimens are uncapped and 400 µL of sample are added. This can be done with Rainin E4 LTS 8-multichannel adjustable 1200 µL pipette (select pipette program 'SAMPLE400'). With the same tips, mix by slowly pipetting up/down 20 times.
- Note: For tube extractions, vortex the tube for 1 min followed by a quick spin step to remove any droplets on the tube cap. The vortexing step enhances the

extraction efficiency, but we recommend it only with a plate format, if the plate can be sealed with pierceable heat seals or each sample is separated by an empty well to avoid cross-contamination problems. The plate can be vortexed in the Tissuelyser for 3 min at 16 Hz and followed by a centrifugation step of the plate in the Megafuge for 2 min at 2,000 g.

- Cover the plate with EasyPierce Seals and heat seal them (175 °C for 2.0 sec) on PX1 PCR plate sealer (Bio-Rad) OR use Clear Adhesive Film (no need for heat sealing with the PX1 PCR plate sealer).
- Heat up water bath to 70 °C. Incubate for 3 min at 70 °C.
- **Note**: heating enhances lysis and activates Proteinase K.
- Place the plate on Magnet Stand-96 for at least 2 min (or until solution becomes clear). If working with tubes, place the tube in the Magnetic Particle Concentrator (MPC). Without disturbing bound beads, carefully remove the entire supernatant (select pipette program 'SN1000').

Step 2: Wash the bound RNA

- Carefully mix the Wash Buffer. Remove the plate or tube from the magnetic stand and add 950 μL Wash Buffer. Mix by slowly pipetting up/down 20 times (select pipette program 'WASH').
- Place plate or tube in the magnetic stand for at least 2 min (or until solution becomes clear). Without disturbing bound beads, carefully remove the entire supernatant (select pipette program 'SN1000').
- Remove the plate or tube from the magnetic stand and add 950 μL 80% ethanol. Mix by slowly pipetting up/down 20 times (select pipette program 'WASH').
- Place the plate or tube on the magnetic stand for at least 2 min (or until solution becomes clear). Without disturbing bound beads, carefully remove the entire ethanol (select pipette program 'SN1000').
- **Note**: it is very important that all the ethanol is gone!
- Leave the plate or tube on the magnet and allow beads to dry for 2 min. If necessary, remove remaining ethanol with a small pipette.

Step 3: Elution of RNA

- Add 50 μL of nuclease-free water to each sample in the plate or tube. Pipette up and down carefully 20 times to ensure all beads are dissolved (select pipette program 'ELUTE').
- Place plate or tube on the magnetic stand for at least 2 min (or until solution becomes clear).
- Carefully transfer 48 μL eluate containing the extracted RNA to a 96-well Elution plate U-bottom (select pipette program 'TRANSFER').
- Continue with RT-qPCR.

3.5. One step RT-qPCR: Same tube Reverse transcription of RNA into cDNA and qPCR

Table SOP3. Master mix for One step RT-qPCR.

**Master Mix for RT-qPCR – Kit 1 (**Luna Probe One-Step Reaction Mix)			
**Stock Concentration**	**Components**	**Final Concentration**	**1× [μL]**
2×	Luna Probe One-Step Reaction Mix	1×	5 μL
20×	Luna WarmStart® RT Enzyme Mix	1×	0.5 μL
6.7 μM primer each	Primer/Probe mix	0. 5μM primer each	0.75 μL

1.7 µM probe		0.13 µM probe	
	template RNA	≤ 1 µg (total RNA)	2.5 µL
	nuclease-free Water		1.25 µL

**Master Mix for RT-qPCR – Kit 2 (**TaqMan™ Fast Virus 1-Step Master Mix)			
**Stock Concentration**	**Components**	**Final Concentration**	**1× [µL]**
4×	Taqman Fast virus 1-step master mix	1×	2.5 µL
6.7 µM primer each<br>1.7 µM probe	Primer/Probe mix	0.5 µM primer each<br>0.13 µM probe	0.75 µL
	template RNA	≤ 1 µg (total RNA)	2.5 µL
	nuclease-free water		4.25 µL

Table SOP4. RT-qPCR program.

Cycle Step	Temperature	Time	Cycles
Priming	25 °C	2 min	
Reverse Transcription	50 °C	20 min	1
Initial Denaturation	95 °C	3 min	1
Denaturation<br>Extension	95 °C<br>58 °C	15 sec<br>10 sec (+plate read)	45×

3.5.1. Preparation of Reaction Mixes

- Thaw Luna Probe One-Step Reaction Mix at room temperature, then place on ice. The enzyme mix should stay at –20 °C until needed/added.

OR:

- Thaw TaqMan™ Fast Virus 1-Step Master Mix on ice.
- Primer/Probe mixes are stored at 4 °C in dark.
- After thawing completely, briefly mix each component by inversion or pipetting. Spin down quickly.
- Prepare the RT-qPCR Reaction Mix for the number of reactions required plus 10% overage on ice. Briefly mix and spin down quickly.
- Aliquot 7.5 µL of assay mix into qPCR plate. Minimize bubbles. Keep plate on cooling block.
- Add 2.5 µL extracted RNA to the qPCR plate. Add also 2.5 µL of the positive RNA control (positive control) or 2.5 µL nuclease-free water (negative control, NTC) in the respectively assigned 384 reaction well. Seal plate with optically transparent film.
- Mix the reaction by tapping on the plate, spin plate briefly to remove bubbles and collect liquid (2 min at 2,000 g).
- start the PCR

3.5.2. CFX Plate Loading

- The transfer of the extracted RNA samples from the 96-well U-bottom plate into the 384-well qPCR plate is shown in Figure 5.

3.5.3. Set Up qPCR Run

- Start the Bio-Rad CFX manger 3.1 program
- open COVID-19-protocol = 'COVID-19-RT-qPCR'
- select COVID-19-plate-Fluorophore choice = 'FAM plate'
- select CFX thermocycler
- start run

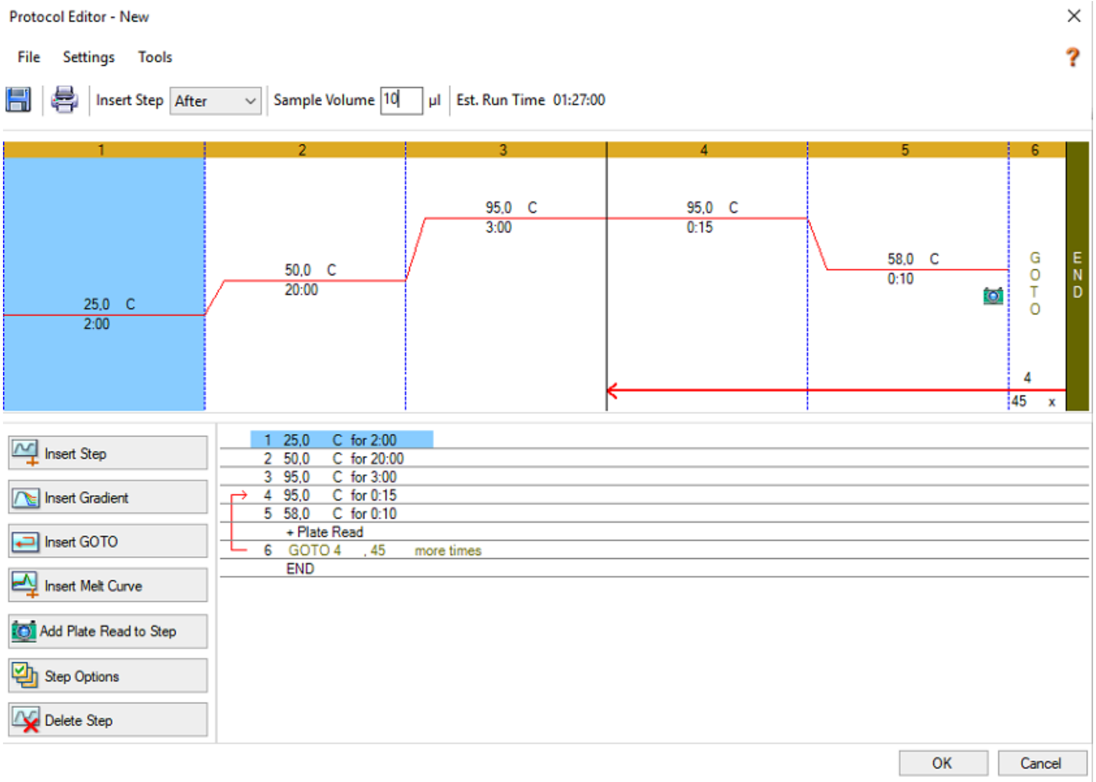

Figure SOP1. CFX RT-qPCR program.

4. Quality Controls

4.1. Experimental Controls

The experimental design incorporates comprehensive controls to monitor all critical steps of both the assay and data interpretation, including specimen quality and RNA extraction efficiency, performance of the reverse transcription and PCR reaction, as well as, pipetting reproducibility. In addition, we use a negative control to monitor contamination. Reproducibility is also warranted by targeting two regions of the COVID-19 *N-gene*.

We also include a negative sample or water as an RNA extraction control in each run.

4.2. Internal Sampling Control

Quality Controls:

- Negative controls: NTC in RT-qPCR (only water)
- Positive controls: Twist Synthetic SARS-CoV-2 RNA Control 1. Positive controls are added in each experiment at 4 different concentrations (10^5, 10^4, 10^3 and 10^2). These positive standards monitor the sensitivity of the reaction and the reproducibility of the measurements, as well as, an evaluation of the PCR efficiency (R-correlation).
- RP target: *RNase P* (low levels of human mRNA) which serves as control for the RNA quality (degradation) in the sample and reverse-transcription efficiency.

4.3. Room Separation and Decontamination

It is absolutely necessary to separate the areas of sample processing, RNA extraction, and RT-qPCR. An amplification product should never be introduced in the area that is intended for extraction and sample material must not be introduced in the area for setting

up the amplification master mix. This is guaranteed since 1) these steps are carried out in different biosafety laminar flow hoods that are cleaned (made free of genetic material) after each process and 2) plates with PCR product stay sealed. Figure 6 shows how the laboratory is separated into four distinct sections. One room is used only for the set-up of the RT-PCR master mix (safety hood 1), the second room is for sample preparation and RNA extraction (safety hood 2), and the last room is for running the PCR reactions in the CFX thermocycler. In case of samples that are stored in non-lysing buffer conditions (NaCl, PBS) the samples are first prepared in safety hood 3 before they are further processed. Rooms 1, 2 and 3 are equipped with an MSC-Advantage™ Class II Biological Safety Cabinets; 1.8 m. Before starting the sample processing, the hoods and pipettes are wiped with DNA-off (Takara) and sterilized under UV light for 10 min. This procedure ensures the destruction of any potential contaminating RNA or DNA. Note that the fast, innate degradation of RNA reduces significantly the potential of contamination from this molecule. To prevent sample contamination 1) each room is used exclusively for the application or technique indicated and 2) the work flow is always unidirectional (indicated by the arrow). Additionally, equipment and consumables are not interchanged between the different laboratory rooms and spaces and accordingly, each room has its own pipettes, tip boxes, gloves and consumables.

5. Data Reporting

5.1. Data Interpretation

Results are interpreted according to the guidelines of the Centers for Disease Control and Prevention approved by the US federal Drug administration for emergency use (https://www.fda.gov/media/134922/download, accessed on 9 June 2021) as followed: where '+' means that amplification with the corresponding primer set was detected (Cq < 45) and '–' means no amplification was detected.

Table SOP5. Result interpretation according to CDC.

N1	N2	RP	Result
+	+	+	positive
+	+	-	positive
+	-	+	inconclusive
-	+	+	inconclusive
+	-	-	inconclusive
-	+	-	inconclusive
-	-	+	negative/not detected
-	-	-	invalid

For samples with inconclusive or invalid results, the whole processing is repeated starting from the extraction (fresh RNA extraction and RT-qPCR). Only negative or positive samples will be uploaded into the KUK mainframe.

5.2. Transfer of Results

The complete procedure (scan of ID probes, experimental master mixes, and position of sample in the 96-well plate for RNA extraction and in the qPCR CFX run) is documented.

Every experiment is documented in a partially automated Excel documentation summary file based on a previously made template to ensure a reproducible analysis and to retrieve experimental procedure information. The file is organized with the following tabs:

read me: instructions for use

samples: Barcode of samples is read in with the barcode reader

RNA extraction und qPCR: Information and instructions for master mixes and reaction components (including lot numbers)

96well und plates visualized: Schematics of sample organization that is automatically created from "**samples**" showing the organization of samples in the 96-well plate for the RNA extraction or the 384-well plate in the qPCR. Pipetting of samples follows this schematic.

384 well cfx export: Data exported from the RT-qPCR run

cfx data: sorted data from the RT-qPCR run

analysis: Analysis of data and automatic assignation of results (positive, negative, inconclusive, invalid). Control reactions are checked and Cq values of the 3 different targets corresponding to the same samples are verified ("true" function).

results: list of the results forwarded to the KUK

The instructions described in the file must be followed. Barcodes of samples are scanned directly into this file. Master mix preparation for the RNA extraction and RT-qPCR are calculated automatically based onto the total samples per experiment and a user-set amount of excess reactions. A visual pipetting scheme for the 96-well and the 384-well plate is automatically created to help with the pipetting. The pipetting scheme is exported as a semi-colon separated .csv file and imported into the Bio-Rad CFX Manager 3.1 to avoid manual data handling. After the RT-qPCR run, the raw data of the run is exported from the Bio-Rad CFX Manager 3.1 and imported into the documentation file. Samples are sorted by sample number and target name (N1, N2 and RP) and manually copied into the analysis tab where a result is assigned to the sample automatically based on the guideline in *Data interpretation.* Instead of differentiating between "inconclusive" and "invalid" results, a new category called "repeat" is used. The tab "results" presents a summary of the results, which is used by the KUK to import this data into their system. LOT numbers, notes, deviations from the protocol and unexpected observations are also documented in this file.

5.3. Storage of Patient's Sample and Data

Patient specimen of positive samples will be stored at –80 °C.

Extracted RNA of positively detected patient samples is transferred from 96-well elution plate to 1.6 mL low-binding tube and will be stored at –80 °C. Those samples are provided with a label containing the sample ID and date of extraction.

6. Warnings or Precautions

6.1. Contamination Prevention

- Lab coats must be worn throughout the whole process and different sets of lab coats are required for each laboratory room. To avoid contamination through the sleeves, disposable sleeves that are exchanged regularly are worn.
- Gloves must be worn during each step of the analysis and must be frequently changed, especially during RNA extraction.
- The working place must be decontaminated with an appropriate cleaning solution (e.g. DNA-off) and the hoods need to be sterilized with 10 min of UV light.
- Never touch the inside of a reaction tube cap.
- To avoid cross-contamination, open only one tube at a time.
- Appropriate micropipette filter tips with aerosol barrier must be used (free of DNase, RNase and human DNA).
- Pipette tips must be changed between liquid transfers (except for master mixes).

- Multichannel pipettes (12- or 24-well channel) are used to avoid confusion of samples or well-misplacement. The use of multichannel pipettes also reduces the number of pipetting steps.
- The samples are always deposited in the same area which is decontaminated after every use.
- Samples are only opened in the BSC hood to reduce the risk of contamination of the personnel.

6.2. Working Safely (Biosafety Level II Conditions)

- To minimise the risk of infection from potentially infectious material, the virus-containing swab is already in inactivating lysis buffer. If not, it is inactivated under laminar air-flow conditions until sample lysis is completed.
- All biological samples are handled and disposed according to the directives for biological safety.
- Avoid direct contact with the biological samples. Aerosols are avoided by the laminar flow hood and samples are handled behind a safety glass.
- Always wear a lab coat and gloves while working with human samples.
- No eating, drinking, or smoking is allowed in the laboratory.
- Wash hands carefully after handling of samples and reagents.
- The personnel are tested on a regular basis to check for infection with the virus.

6.3. General Precautions

- This set-up is for *in vitro* diagnostic use only and should only be used by trained personnel with qualifications in molecular biology.
- Upon arrival, check the kit components for damage. Do not use damaged kit components, as their use may lead to poor kit performance.
- Check the expiration dates of the reagents.
- Do not use expired reagents (e.g. RT-qPCR kit).
- Pipetting of small amounts of liquid in the microliter range is a challenge. Pipetting is monitored via labelled tips and multichannel. Revision of pipetting volume before each pipetting step. The use of electronic pipettes ensures the accurate and reproducible pipetting of volumes.
- Pipetting errors during preparation of the components like master mixes or during RNA extraction is limited with the use of multichannel pipettes and electronic pipettes
- PCR program on thermocycler is pre-programmed to avoid the wrong choice of PCR conditions (program template with input data of sample IDs).

6.4. Contamination Containment

- Regular checks for contamination with NTC are performed in each experiment.
- In case of contamination, all open aliquots will be discarded and a new aliquot will be taken. This includes all the reagents for RNA extraction, as well for the RT-qPCR reaction.
- Decontamination is carried out by the treatment of the hoods with UV light, wiping the hood surfaces and the pipettes with DNA-off, exchanging tip boxes, lab coats and arm sleeves.
- Door handles and machine surfaces will also be wiped with DNA-off.
